# Boot Jelly and Shirt Coffee

**Published:** 1875-01

**Authors:** 


JELLY FROM OLD BOOTS.—The reader may stare, but Science smiles superior and asserts very emphatically that a toothsome delicacy can be made from a dilapidated foot covering. Some time ago, Dr. Vander Weyde, of this city, regaled some friends not merely with boot jelly, but with shirt coffee, and the repast was pronounced by all partakers excellent. The doctor tells us that he made the jelly by first cleaning the boot, and subsequently boiling it with soda, under a pressure of about two atmospheres. The tannic acid in the leather, combined with salt, made tennate of soda, and the gelatin rose to the top, whence it was removed and dried. From this last, with suitable flavoring material, the jelly was readily concocted. The shirt coffee, which we incidentally mentioned above, was sweetened with cuff and collar sugar, both coffee and sugar being produced in the same way. The linen (after, of course, washing) was treated with nitric acid, which, acting on the lignite contained in the fiber, produced glucose or grape sugar. This, roasted, made an excellent imitation coffee, which an addition of unroasted glucose readily sweetened.—*Scientific American.*

